# Effects of *Artemisia argyi* flavonoids on growth performance and immune function in broilers challenged with lipopolysaccharide

**DOI:** 10.5713/ab.20.0656

**Published:** 2021-01-01

**Authors:** Shuo Yang, Jing Zhang, Yang Jiang, Yuan Qing Xu, Xiao Jin, Su Mei Yan, Bin Lin Shi

**Affiliations:** 1 College of Animal Science, Inner Mongolia Agricultural University, Hohhot 010018, China

**Keywords:** Flavonoid, Immunomodulation, Lipopolysaccharide Challenge, Broiler

## Abstract

**Objective:**

This research aimed to study the effects of *Artemisia argyi* flavonoids (AAF) supplemented in diets on the growth performance and immune function of broiler chickens challenged with lipopolysaccharide (LPS).

**Methods:**

A total of one hundred and ninety-two 1-d-old broiler chicks were assigned into 4 treatment groups, which were, respectively, fed a basal diet (control), fed a diet with 750 mg/kg AAF, fed a basal diet, and challenged with LPS, fed a diet with 750 mg/kg AAF, and challenged with LPS. Each treatment had six pens with 8 chicks per pen. On days 14, 16, 18, 20 (stress phase I) and 28, 30, 32, 34 (stress phase II), broilers were injected with LPS (500 μg/kg body weight) or an equivalent amount of saline.

**Results:**

The results demonstrated that dietary AAF significantly improved the body weight (d 21) and alleviated the decrease of average daily gain in broilers challenged with LPS on d 21 and d 35 (p<0.05). Dietary AAF increased bursa fabricius index, and dramatically attenuated the elevation of spleen index caused by LPS on d 35 (p<0.05). Furthermore, serum interleukin-6 (IL-6) concentration decreased with AAF supplementation on d 21 (p<0.05). Diet treatment and LPS challenge exhibited a significant interaction for the concentration of IL-1β (d 21) and IL-6 (d 35) in serum (p<0.05). Additionally, AAF supplementation mitigated the increase of IL-1β, IL-6 in liver and spleen induced by LPS on d 21 and 35 (p<0.05). This study also showed that AAF supplementation significantly reduced the expression of *IL-1β* (d 21) and nuclear transcription factor kappa-B p65 (d 21 and 35) in liver (p<0.05), and dietary AAF and LPS treatment exhibited significant interaction for the gene expression of *IL-6* (d 21), toll like receptor 4 (d 35) and myeloid differentiation factor 88 (d 35) in spleen (p<0.05).

**Conclusion:**

In conclusion, AAF could be used as a potential natural immunomodulator to improve growth performance and alleviate immune stress in broilers challenged with LPS.

## INTRODUCTION

In the past few decades, the broiler farming industry has rapidly grown to meet the increasing requirement of the growing human population and is still growing [1]. However, the broilers are prone to immunologic stress due to unsuitable environmental conditions (such as virus attack, nutrient deficiency, and high stock density) [2]. Earlier studies showed that immunosuppressive diseases reduced the average daily weight gain and seriously affected the thymus and bursal index of broilers, leading to health impairment [3]. In addition, the endocrine system and metabolism of the animal could be affected by immune stress, which might result in decreased growth performance [4]. Consequently, from the above reasons, novel types of feed additives that can protect animals to allay immune stress and improve performance are in high demand.

Plant flavonoids as naturally occurring plant secondary metabolites are receiving noticeable attention due to their potential great multidimensional biological functions. In nature, flavonoids are widely found in the roots, stems, leaves, fruits of plants, and were classified into six groups according to hydroxylation patterns and the unsaturation degree of their chemical backbones [5]. Reports showed that plant flavonoids acted as mild immunomodulators, up-regulated the adaptive immune responses that occurred in intestinal inflammation, and then improved intestinal digestion efficiency [6]. Dietary inclusion of flavonoids could ameliorate antioxidative status when stimulated by lipopolysaccharide (LPS) in broilers, as well as enhance the quality of chicken meat [7]. Furthermore, flavonoids casticin and chrysosplenol D from *Artemisia annua* L. suppressed the expression of inflammatory factors in mice [8].

*Artemisia argyi*, a kind of traditional Chinese herbal medicine, has been proven to contain numerous flavonoids besides polysaccharides and volatile oils, and these materials have been actively studied in animal production [9]. These findings were similar to ours which showed the extract of *Artemisia argyi* contained polysaccharides, flavonoids, etc., and completed a preliminary study on the effect of the extract of *Artemisia argyi* on broiler chickens [10]. However, there were still not enough studies on the regulation action of *Artemisia argyi* flavonoids (AAF) on the immune system in broilers. Therefore, the objective of this experiment was to investigate the effects of flavonoids extracted from the *Artemisia argyi* on the growth performance and immune function of broilers using an immune stress model induced by LPS.

## MATERIALS AND METHODS

The animal experiment was performed in accordance with the animal care and use guideline (GB/T 35892-2018).

### Plant material and extracts

Fresh *Artemisia argyi* was collected from Hohhot (Inner Mongolia, China) in July. The aerial portion of *Artemisia argyi* was obtained and dried at room temperature in the shade. The AAF was extracted from *Artemisia argyi* powder as previously described [11]. Briefly, 40 g of the dried parts were powdered and extracted with 800 mL 75% ethanol (solid: ethanol = 1:20) for 4 h at 70°C. The extracting solution was filtered and concentrated using a vacuum evaporator, and then lyophilized to obtain the crude extract which was stored at −20°C until used in the experiment. Total flavonoid content was determined by the colorimetric method using rutin as a standard according to the method described previously [11]. Results were expressed as milligram rutin equivalents (RE)/gram extract and the content of total flavonoid in the extracts was 367.9 mg RE/g. These extracts were denominated as AAF and used in the broiler feeding experiment.

### Animals and housing

A total of one hundred and ninety-two 1-day-old commercial Arbor Acres broiler chickens with a mean initial weight of 47.04±0.02 g (mean±standard deviation) were purchased from a local hatchery. All the chicks were housed in temperature-controlled single-layer wire cages. The temperature was maintained at 32°C to 34°C in the first week and gradually reduced by 2°C to 3°C each week to a final temperature of 22°C at week 4, then maintained at 22°C until the end of the experiment. The relative humidity in the house varied from 60% to 65%. In addition, the lighting scheme of 23 L:1 D (23 h of lighting and 1 h of dark per day) was provided during the first week, followed by 14 L:10 D from day 8 to day 35, and thereafter 23 L:1 D until day 42. All birds could consume feed and water *ad libitum* during the rearing period. The starter (1 to 21 days of age) and grower (22 to 42 days of age) diets were fed in mash form and were based on corn-soybean meal. The essential nutrients provided in diets conform to the nutrient recommendations as NRC (1994) of broiler chickens and meet nutrients recommendations of Feeding Standard of Chicken, China (NY/T 33-2004) (Chinese Ministry of Agriculture 2004) (Table 1).

### Experimental design

This experiment was performed based on a 2×2 factorial design with LPS (injection or no injection) and dietary AAF (supplementation or no supplementation). According to our previous studies, the addition of 750 mg/kg AAF to basal diet could improve the growth performance, increase immunity and antioxidant capacity of broiler chickens, therefore, the supplementation dose of AAF in diet was 750 mg/kg in the current experiment. The birds were randomly assigned into 4 dietary treatments with 6 replications of 8 birds in each replication, and the four treatments were as follows: i) basal diet and injection with saline (0.9%); ii) basal diet and injection with LPS (500 μg/kg body weight [BW]); iii) basal diet with 750 mg/kg AAF and injection with saline (0.9%); iv) basal diet with 750 mg/kg AAF and injection with LPS (500 μg/kg BW). The experimental period was divided into the adaptation period (days 0 to 14), stress phase I (days 15 to 28, including 7-day LPS injection period, 7-day recovery period), and stress phase II (days 29 to 42, including 7-day LPS injection period, 7-day recovery period). At days 14, 16, 18, 20 instress phase I and at days 28, 30, 32, 34 instress phase II, LPS or equivalent saline were injected into the intraperitoneal of broilers.

### Growth performance

The chickens and feed residues in each pen were weighed weekly, and the average BW, average daily gain (ADG), average daily feed intake (ADFI), and the ratio of feed to gain (F:G) were calculated during the experimental.

### Sample collection

Blood was collected at four time points. On d 21 (stress phase I), d 28 (recovery period I), d 35 (stress phase II) and d 42 (recovery period II) of age, respectively, 10 mL of blood sample was collected from the wing vein of one broiler chicken randomly selected from each replicate pen. Serum samples were obtained by centrifuging at 3,000×g for 15 min at 4°C and then immediately stored at −20°C until further analysis. In addition, at the end of each LPS injection period (d 21 and d 35), with following blood collection, the broiler chicken was killed by exsanguination, subsequently the tissue sample of liver and spleen was cut and frozen in liquid nitrogen, and then stored at −80°C for RNA extraction. Another tissue sample of the liver and spleen was minced and homogenized (10% w/v) in saline, then centrifuged at 3,000×g for 10 min at 4°C, and then the supernatant was collected and stored at −20°C for the determination of relevant immune indicators.

### Organ index

After slaughter, the organs including thymus, spleen, bursa, and liver were removed and weighed after stripping fat and irrelevant tissues. And then the organ index was calculated by the following formula:

Organ index=organ weight (g)/body weight (kg)

### Cytokine content

The concentrations of interleukin-1β (IL-1β) and interleukin-6 (IL-6) in serum and tissue homogenate supernatant were determined using commercially available enzyme-linked immunosorbent assay test kits (Nanjing Jiancheng Biotechnology Institute, Nanjing, China) according to the method suggested in respective kit manuals.

### Real-time polymerase chain reaction

Total RNA in the liver and spleen tissues was extracted using TRIzol reagent (Invitrogen, Carlsbad, CA, USA), and then the isolated RNA was quantitatively and qualitatively determined as described in the previous report [12]. RNA was reverse-transcribed into cDNA using the PrimeScript RT reagent Kit after removing genomic DNA contamination (Takara Bio Inc., Otsu, Japan) according to the manufacturer’s instructions. Quantification of cDNA transcript was performed using qPCR TB Green Kit with the gene-specific primer on the LightCycler 96 real-time PCR system. The target genes and their primer sequences are listed in Table 2, which were designed and synthesized by Shanghai Sangon Biotech (Shanghai, China). β-Actin was used as a reference gene and the relative transcript quantities in the mRNA expression levels were calculated using the 2^−ΔΔCT^ method.

### Statistical analysis

All data were analyzed using the general linear model procedure of SAS 9.2 software as 2×2 factorial arrangement with dietary treatments of AAF and LPS challenge status as main effects and were analyzed for the main effects and interactions between AAF and LPS. The data is presented as mean along with pooled standard error of means (mean±standard error of the mean), and p<0.05 was considered to be statistically significant.

## RESULTS

### Growth performance

The effect of dietary AAF on the growth performance (BW, ADG, ADFI, and F:G) of broilers challenged with LPS is shown in Table 3. Intraperitoneal injection with LPS significantly decreased BW of the broilers during all the experimental period (p<0.05). But dietary AAF significantly increased the BW of the broiler chickens on d 21 (p<0.01) regardless of LPS challenge, compared with basal diet groups. In addition, in the broilers challenged by LPS, the ADG and ADFI were significantly decreased (p<0.01) compared with those treated with saline (d 15 to 21 and d 29 to 35). There was a significant interaction between LPS and AAF on the ADG of the broiler chickens at d 29 to 35 (p<0.05). But the results showed that supplementation with AAF in basal diet and injection with LPS did not affect F:G throughout the experiment (p>0.05).

### Organ index

The effects of AAF on the relative weight of organs in broilers challenged with LPS are shown in Table 4. LPS challenge significantly increased spleen index on d 21, d 35, and thymus index on d 21 compared with no LPS challenge (p< 0.05). Furthermore, diet and LPS exhibited significant interaction for spleen index on d 35 (p = 0.05). In addition, diet supplemented with AAF increased the bursa fabricius index of chickens compared with the basal diet on d 35 (p<0.05). No effect on liver index was detected with supplementing AAF in diet during the LPS challenge phase. Diet and LPS exhibited no significant interaction (p>0.05).

### Serum cytokines

The effects of AAF on cellular immunity of broilers challenged with LPS are presented in Table 5. In comparison with saline injection, LPS injection increased serum concentrations of IL-1β and IL-6 on d 21 and d 35 (p<0.05). The diet supplemented with AAF significantly decreased the serum concentration of IL-6 compared with basal diet on d 21 (p = 0.04). Moreover, a significant interaction between AAF and LPS challenge was observed for IL-1β (d 21) and IL-6 (d 35) (p<0.05). Furthermore, diet and LPS challenge exhibited a significant interaction for *IL-6* in recovery period (on d 28) (p<0.05).

### Liver immune cytokines

The measurement results of the immune cytokines in liver are shown in Table 6. Dietary supplementation of AAF significantly decreased IL-1β and IL-6 concentrations in liver of broilers compared with basal diet on d 35 (p<0.01). During LPS challenged phases (d 21 and d 35), LPS challenge significantly increased the concentrations of IL-1β and IL-6 in liver (p<0.05). Furthermore, dietary AAF and LPS challenge exhibited significant interaction for IL-1β on d 21 and IL-1β, IL-6 on d 35, respectively (p<0.05).

### Spleen immune cytokines

The data of spleen immune cytokines in broilers are shown in Table 7. Compared with unchallenged chickens, the broilers challenged with LPS showed an increase in the contents of IL-1β and IL-6 in spleen on d 21 and d 35 (p<0.05). Interestingly, dietary supplementation with AAF reduced the IL-1β and IL-6 contents compared with basal diet (p<0.05) on d 21 and 35. Additionally, LPS and AAF showed a significant interaction in IL-1β content on d 35 (p = 0.03).

### Liver gene expression

**Figure 1:**
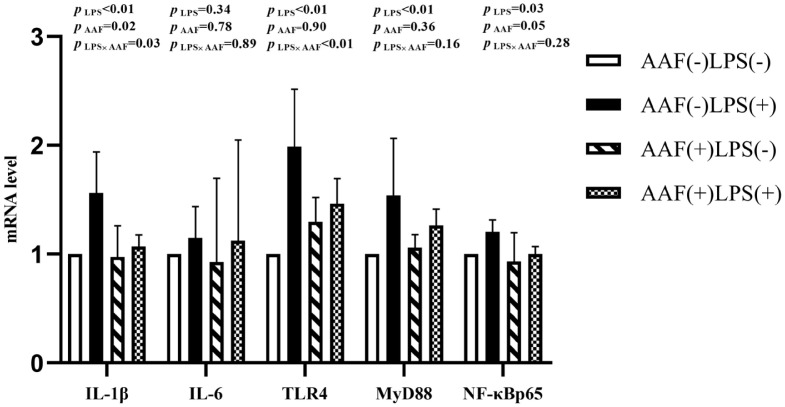
Effects of fed diets with or without 750 mg/kg AAF on inflammation-related genes expression in the liver of broilers challenged by LPS on day 21. AAF, *Artemisia Argyi* flavonoids; LPS, lipopolysaccharide; IL-1β, interleukin-1β; IL-6, interleukin-6; TLR4, toll like receptor 4; MyD88, myeloid differentiation factor 88; NF-κB p65, nuclear transcription factor kappa-B p65. Less than 0.05 p value was taken to indicate significance.

**Figure 2:**
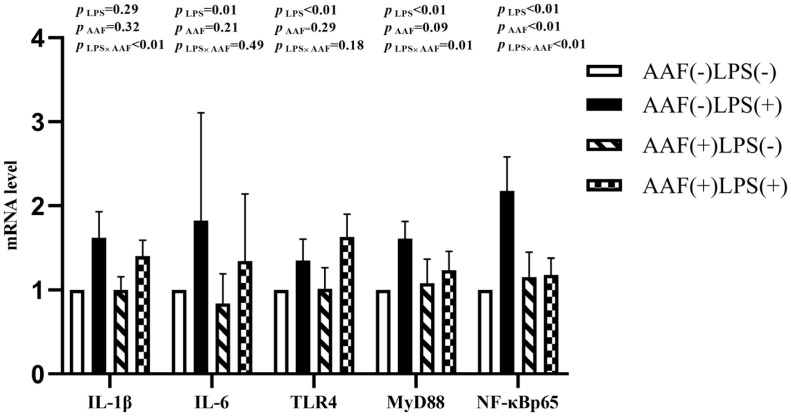
Effects of fed diets with or without 750 mg/kg AAF on inflammation-related genes expression in the liver of broilers challenged by LPS on day 35. AAF, *Artemisia Argyi* flavonoids; LPS, lipopolysaccharide; IL-1β, interleukin-1β; IL-6, interleukin-6; TLR4, toll like receptor 4; MyD88, myeloid differentiation factor 88; NF-κB p65, nuclear transcription factor kappa-B p65. Less than 0.05 p-value was taken to indicate significance.

Figure 1 shows the effects of different treatments on the gene expression in liver. LPS challenge increased the mRNA expression of *IL-1β*, toll like receptor 4 (*TLR4*), myeloid differentiation factor 88 (*MyD88*), and nuclear transcription factor kappa-B (*NF-κB*) p65 in liver compared with saline injection on d 21 (p<0.05). However, supplementation of AAF in diet reduced the mRNA expression of *IL-1β* and *NF-κBp65* compared with basal diet (p<0.05). Moreover, dietary AAF and LPS challenge exhibited a significant interaction for the mRNA expressions of *IL-1β* and *TLR4* in liver of broilers on d 21 (p<0.05). On d 35, compared with unchallenged chickens, those challenged with LPS elevated the mRNA expression of *IL-6*, *TLR4*, *MyD88*, and *NF-κBp65* (p<0.05, Figure 2). However, dietary supplementation with AAF could decrease the mRNA expression of *NF-κBp65* in liver even though under LPS challenge condition, when compared with un-supplement with AAF (p<0.01). In addition, significant interactions between AAF and LPS were observed for the expression of *IL-1β*, *MyD88*, and *NF-κBp65* on d 35 (p<0.05).

### Splenic gene expression

**Figure 3:**
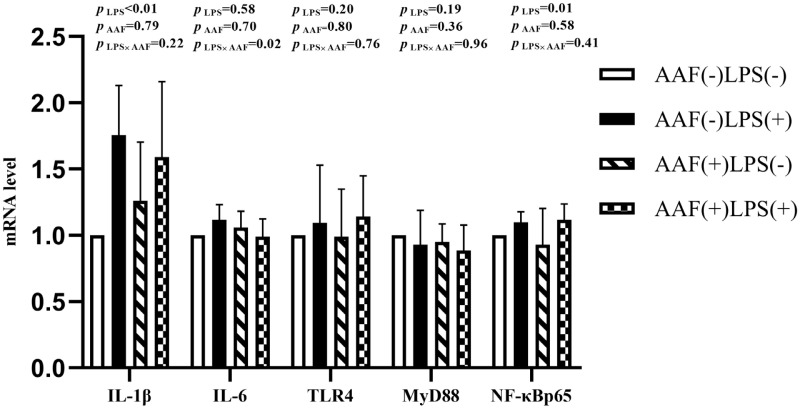
Effects of fed diets with or without 750 mg/kg AAF on inflammation-related genes expression in the spleen of broilers challenged by LPS on day 21. AAF, *Artemisia Argyi* flavonoids; LPS, lipopolysaccharide; IL-1β, interleukin-1β; IL-6, interleukin-6; TLR4, toll like receptor 4; MyD88, myeloid differentiation factor 88; NF-κB p65, nuclear transcription factor kappa-B p65. Less than 0.05 p-value was taken to indicate significance.

**Figure 4:**
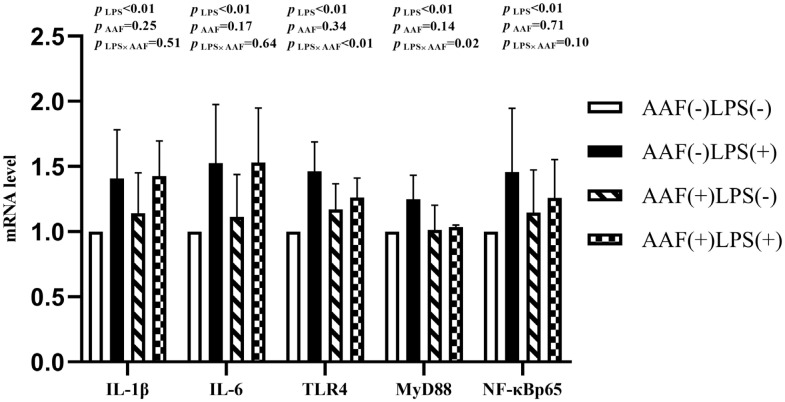
Effects of fed diets with or without 750 mg/kg AAF on inflammation-related genes expression in the spleen of broilers challenged by LPS on day 35. AAF, *Artemisia Argyi* flavonoids; LPS, lipopolysaccharide; IL-1β, interleukin1β; IL-6, interleukin-6; TLR4, toll like receptor 4; MyD88, myeloid differentiation factor 88; NF-κB p65, nuclear transcription factor kappa-B p65. Less than 0.05 p-value was taken to indicate significance.

The splenic gene expression data from the broilers are shown in Figure 3. On d 21, intraperitoneal injection of LPS up-regulated the mRNA expression of *IL-1β* and *NF-κBp65* in spleen compared to injection of saline (p<0.01). However, supplementation with AAF in diet did not affect *IL-1β*, *IL-6*, *TLR4*, *MyD88*, and *NF-κBp65* mRNA expression in response to LPS (p>0.05). Diet and LPS challenge exhibited a significant interaction for *IL-6* expression on d 21 (p<0.05). On d 35, broilers in LPS challenge group exhibited higher mRNA levels of *IL-1β*, *IL-6*, *TLR4*, and *NF-κBp65* in spleen than those in the unchallenged group (p<0.01, Figure 4). Furthermore, the interaction between dietary AAF treatment and LPS challenge was significant for the expression of *TLR4* and *MyD88* in spleen (p<0.05).

## DISCUSSION

LPS is composed of lipids and polysaccharides, which is the primary cell wall component of the outer membrane of gram-negative bacteria. Intraperitoneal or intravenous injection of LPS can induce infection and cause inflammation in broiler chickens, so it is a widely used model for research animal immune stress [13]. Immune stress caused by LPS challenge seriously influences ADFI and ADG of broiler chickens [14]. The present study showed that BW, ADG, and ADFI were decreased when a broiler chicken was injected with LPS, which consistent with a previous study [2]. One primary cause is that the hypothalamic-pituitary-adrenal axis is activated, and signal molecule receptors about appetite are stimulated, thence anorexia response appears; another reason is that pro-inflammatory cytokines are produced under immune stress, resulting in the repartition of nutrients, and an increasing in the catabolic activities in the process of animal growth [15].

Plant flavonoids were confirmed to have antioxidant activities and immunomodulatory effects [7]. In a chicken feeding study [16], dietary 200 mg/kg of flavonoid baicalein not only increased ADG, feed conversion ratio, but also increased the ratio of CD3+/CD4+, and the concentration of interferon gamma of broiler chickens. Furthermore, another study [17] indicated that dietary genistein supplementation in diet significantly improved growth performance and affected immunological responses in broiler chicks. It is generally recognized that flavone can promote the combination of growth hormone and hepatic growth hormone receptor and then induce growth promotion [18]. The present study showed that AAF increased the BW and ADG of broilers compared with basal diet on d 21. Moreover, supplement with AAF in diet could alleviate immune stress and increase the ADG and ADFI in broilers stimulated by LPS on d 21 and 35, and the experiment confirmed that dietary AAF treatment and LPS challenge significantly impacted ADG of the broilers on d 35. Similar to the present study [19], dietary flavones from sea buckthorn fruits quadratically improved ADFI, ADG, and BW in broilers. Therefore, our results suggested that dietary AAF might exert a protective effect on the growth of the broilers via to ameliorate the adverse.

The thymus, spleen, and bursa are the vital immune organs because they can participate in immune regulation and reflect the immune status of poultry. In recent decades, flavonoids from medicinal plants attracted much attention because of their potential immunomodulatory function in living systems. Previous reports showed that flavonoids as feed additives significantly increased the sizes of thymus, bursa, and spleen [20]. The data from the current study indicated that injection with LPS increased spleen index and thymus index in the broilers, which might be associated with the acute inflammation causing the greater activity of immune organs, and then the emergency need for cytokine and antibodies synthesis during the response. That is consistent with a recent *in vivo* study which revealed that injection with LPS could increase spleen index and promote proinflammatory cytokines secretion of broiler chicks [21]. However, the results from our current study demonstrated that the interaction between dietary AAF and LPS challenge significantly impacted spleen index of broilers. The present results suggested the possibility that supplementary flavonoids promoted the phagocytic activity of spleen macrophages and the efficiency of peritoneal leukocytes, therefore, alleviated the damage of visceral organs caused by pathogenic bacteria and viruses. In addition, dietary AAF increased the relative weight of bursa fabricius. These results were not consistent with previous study [22], which reported that dietary bamboo leaf flavonoid did not affect the weights of immune organs (spleen, thymus, and bursa fabricius). The different responses of flavonoids to the immune organ in these reports may be attributed to different types or dosages of flavonoids.

LPS injection elevates the levels of inflammatory cytokines, resulting in the imbalance between pro-inflammatory and anti-inflammatory systems and contributing to immune stress of cells and tissues. Several researchers reported that the contents of pro-inflammatory cytokines including IL-1β, IL-6, and tumor necrosis factor alpha (TNF-α) increased after LPS challenge in chicks [23]. The present study indicated that intraperitoneal injection with 500 μg LPS per kg BW increased the contents of IL-1β andIL-6 in the serum of broilers at d 21 and 35. This result suggest that the inflammation model was successfully constructed. Excessive production of pro-inflammatory factors would cause the nutrients used to maintain growth and bone deposition to be used to maintain the stability of the immune system, and induce anorexia [24], thereby reduce the growth performance of broilers. However, AAF supplementation in diet dramatically decreased IL-1β content on d 21 regardless of LPS challenge. Furthermore, there was a significant interaction between dietary AAF and LPS for IL-1β, IL-6 on d 21 and 35. Results obtained in the present study were consistent with findings of Wu et al [25], who reported supplementation of procyanidin modulated the *in vivo* secretion of IL-1β, IL-6, and INF-γ in LPS-challenged broilers. Hence, it could be speculated that the flavonoids can induce the normal secretion of pro-inflammatory cytokines by modulating the functions of macrophages. In addition, the present results indicated that dietary AAF alleviated the negative effects of immune stress by regulating the production of pro-inflammatory cytokines in serum of broilers.

As an important organ involved in nutrient metabolism, detoxification, and secretion in livestock, the liver is susceptible to damage by endotoxins and live bacteria. Moreover, liver is the site for protein synthesis and is significantly important for health maintenance [25]. In the present study, the contents of IL-1β, IL-6 and relative weight were increased in the liver of broilers challenged by LPS. The results indicated that the intraperitoneal injection of LPS could induce inflammatory reactions in broilers and cause immune damage in liver. But according to the results of this experiment, we found that supplementation with AAF alleviated the increase of IL-1β, IL-6 concentration induced by LPS challenge and there was an AAF×LPS interaction for liver immune cytokines, which suggested that AAF has an important role in recovering the physiological balance in broilers. In chickens, the spleen acts as a vital secondary lymphoid tissue, and its health status reflects systemic immune function, but it is easily damaged when challenged with pathogenic infections [26]. A study [27] found that LPS challenge induced inflammatory injury as exhibited by decreased cell proliferation and enhanced apoptosis in the spleen of young chicks. In the current investigation, we observed an increase in the contents of IL-1β and IL-6 in spleen following LPS stimulation. As discussed before, flavonoids possess the potential to alleviate immune stress. In our study, we found that AAF supplementation was beneficial in protecting the spleen from immune damage as evidenced by alleviated production of inflammatory cytokine, which ultimately improved anti-inflammatory capacity of broilers.

**Figure 5:**
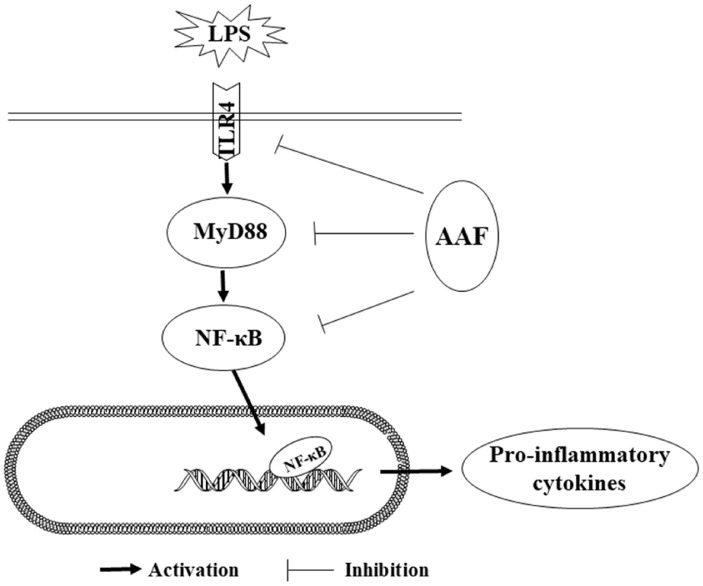
Anti-inflammatory mechanism of AAF is associated with suppressing the TLR4 mediated NF-κB signaling pathway induced by LPS. AAF, *Artemisia Argyi* flavonoids; TLR4, toll like receptor 4; NF-κB, nuclear transcription factor kappa-B; LPS, lipopolysaccharide.

One possible mechanism for the immunomodulatory effect of AAF might be through nuclear factor-kappa B (NF-κB) pathways to alleviate immune damage. Several studies indicated that NF-κB was a critical transcription factor in the Rel family, which was responsible for the transcription of genes involved in immune responses [28]. Generally, NF-κB binding to inhibitor of NF-κB (I-κB) constituted a cytoplasmic complex and maintained a silent state under unstimulated conditions. When chickens were exposed to immune stress conditions, TLR4 combining with MyD88 in cell-initiated activation of the downstream NF-κB signaling pathways and released pro-inflammatory cytokines. TLR4 can combine with myeloid differentiation protein-2 (MD-2) on cell surfaces and form the critical pattern recognition receptor for LPS in host defense. Our data showed that LPS challenge increased mRNA abundances of *TLR4*, *MyD88*, *NF-κBp65*, *IL-1β*, and *IL-6* in the liver of broilers. Moreover, LPS challenge increased the mRNA expression of *IL-1β*, *IL-6*, *NF-κBp65*, *MyD88*, and *TLR4* in the spleen, which was consistent with the previous study [29], indicating that the NF-κB pathway was activated by LPS. It was reported that the flavonoids isolated from plants could downregulate NF-κB signal transduction pathway, reduce the pro-inflammatory mediators such as *IL-1β* and *TNF-α*, thereby, inhibit inflammatory responses. Previous research [30] showed that the NF-κB activity and I-κB phosphorylation were modulated by flavonoids and the symptoms of colitis in mice were decreased. Moreover, when RAW264.7 cells were stimulated by LPS, the level of p-NF-κB and contents of IL-1β, TNF-α were notably increased in the culture supernatants, whereas pretreatment of flavonoids (50 μg/mL) inhibited the nucleus translocation of p-NF-κB and reduced the mRNA expression of *TNF-α* and *IL-6*. Similar to these results, our research found that flavonoids from *Artemisia argyi* could decrease the mRNA expression of *IL-1β*, *NF-κBp65* in liver of broilers. Furthermore, diet and LPS treatment exhibited a significant interaction for the gene expression of *IL-6*, *TLR4*, and *MyD88* in spleen. These results indicated that AAF played an important role in counteracting the immune stress via activating NF-κB signaling pathway. Mechanistically, our results showed that AAF significantly inhibited the activity of *TLR4* and *MyD88* in the liver and spleen of broilers with inflammation induced by LPS, which suggested that the NF-κB signaling pathway was suppressed and inflammatory response was alleviated (Figure 5).

## CONCLUSION

In summary, our results showed that the immune stress induced by LPS could decrease the growth performance and cause inflammatory response of broilers. However, dietary supplementation with AAF could alleviate the decrease of growth performance and immune function in broilers challenged by LPS. The protective mechanism might involve the NF-κB signaling pathway. Hence, the AAF might be a potential natural feed additive but needs to be verified by further experiments.

## Figures and Tables

**Table 1:** Composition and nutrient level of the basal diet (air-dry basis)

Items	1 to 21 days of age	22 to 42 days of age
Ingredients		
Corn	52.50	58.80
Soybean meal	40.00	33.80
Soybean oil	3.00	3.00
Dicalcium phosphate	1.90	1.80
Limestone	1.08	1.22
Salt	0.37	0.37
L-lysine	0.05	0.03
DL-methionine	0.19	0.07
Premix^1)^	0.80	0.80
Choline	0.11	0.11
Total	100.0	100.0
Nutrient levels^2)^		
Metabolizable energy/(MJ/kg)	12.42	12.62
Crude protein	21.77	19.65
Calcium	1.00	1.02
Available phosphorus	0.44	0.42
Lys	1.34	1.15
Met	0.55	0.40
Cys	0.40	0.36

1) Premix provided the following per kilogram of diet: vitamin A 9,000 IU, vitamin D_3_ 3,000 IU, vitamin E 26 mg, vitamin K_3_ 1.20 mg, vitamin B 13.00 mg, vitamin B_2_ 8.00 mg, vitamin B_6_ 4.40 mg, vitamin B_12_ 0.012 mg, nicotinic acid 45 mg, folic acid 0.75 mg, biotin 0.20 mg, choline 1,100 mg, calcium pantothenate 15 mg. Fe 100, mg, Cu 10 mg, Zn 108 mg, Mn 120 mg, I 1.5 mg, Se 0.35 mg.

2) Crude protein was measured value, while others were all calculated values.

**Table 2:** Genes and their primer sequences

Gene name	Gene Bank NO.	Primer sequence	Fragment size (bp)
*β-Actin*	NM_205518	F-GCCAACAGAGAGAAGATGACAC R-GTAACACCATCACCAGAGTCCA	118
*IL-1β*	NM_204524	F-CAGCCTCAGCGAAGAGACCTT R-ACTGTGGTGTGCTCAGAATCC	84
*IL-6*	HM179640	F-AAATCCCTCCTCGCCAATCT R-CCCTCACGGTCTTCTCCATAAA	106
*NF-κB p65*	D13721	F-CAGCCCATCTATGACAACCG R-CAGCCCAGAAACGAACCTC	151
*TLR4*	NM_001030693	F-TTCAGAACGGACTCTTGAGTGG R-CAACCGAATAGTGGTGACGTTG	131
*MyD88*	NM_001030962	F-CCTGGCTGTGCCTTCGGA R-TCACCAAGTGCTGGATGCTA	198

F, forward primer; R, reverse primer; *IL-1β*, interleukin-1β; *IL-6*, interleukin-6; *NF-κB p65*, nuclear transcription factor kappa-B p65; *TLR4*, toll like receptor 4; *MyD88*, myeloid differentiation factor 88.

**Table 3:** Effects of *Artemisia Argyi* flavonoids on growth performance of broilers challenged with lipopolysaccharide

Item	LPS(−)^1)^		LPS(+)^1)^		SEM	p-value		
		
	AAF(−)^2)^	AAF(+)^2)^	AAF(−)^2)^	AAF(+)^2)^		AAF	LPS	AAF×LPS
BW (kg)								
14 d	0.30	0.29	0.29	0.29	0.01	-	-	-
21 d	0.61	0.65	0.59	0.62	0.01	<0.01	0.01	0.80
28 d	1.07	1.10	1.03	1.03	0.01	0.25	<0.01	0.34
35 d	1.60	1.63	1.51	1.54	0.02	0.35	0.02	0.88
42 d	2.15	2.17	2.02	2.04	0.04	0.62	0.02	0.96
ADG (g/d)								
1–14 d	17.84	17.78	17.16	17.32	0.47	-	-	-
15–21 d	45.88	49.39	41.08	44.62	0.91	<0.01	<0.01	0.99
22–28 d	64.01	65.10	62.69	61.82	1.43	0.94	0.12	0.50
29–35 d	73.36	79.425	66.40	69.42	1.68	0.77	<0.01	0.04
36–42 d	71.85	76.99	72.70	71.01	2.13	0.48	0.30	0.18
ADFI (g/d)								
1–14 d	23.41	22.84	23.63	23.01	0.80	-	-	-
15–21 d	64.68	67.34	61.30	62.14	1.22	0.28	0.02	0.57
22–28 d	104.43	105.55	106.49	99.54	2.60	0.34	0.51	0.89
29–35 d	130.30	136.79	122.80	123.9	2.80	0.52	<0.01	0.67
36–42 d	151.88	155.00	161.40	150.13	4.20	0.28	0.48	0.09
F:G								
1–14 d	1.24	1.24	1.28	1.28	0.03	-	-	-
15–21 d	1.36	1.32	1.43	1.37	0.04	0.31	0.16	0.88
22–28 d	1.52	1.55	1.64	1.58	0.05	0.76	0.18	0.48
29–35 d	1.81	1.76	1.72	1.88	0.05	0.38	0.81	0.09
36–42 d	2.05	1.98	1.99	2.14	0.09	0.65	0.58	0.22

Data represent means from 6 replicates per treatment.

AAF, *Artemisia Argyi* flavonoids; LPS, lipopolysaccharide; SEM, standard error of the mean; BW, body weight; ADG, average daily gain; ADFI, average daily feed intake; F:G, feed:gain.

1) LPS(−),birds were intraperitoneally injected with saline; LPS(+), birds were intraperitoneally injected with LPS.

2) AAF(−), basal diet; AAF(+), basal diet supplemented with 750 mg/kg AAF.

**Table 4:** Effects of *Artemisia Argyi f*lavonoids on relative weight of organs in broilers challenged with lipopolysaccharide

Item (g/kg)	LPS(−)^1)^		LPS(+)^1)^		SEM	p-value		
		
	AAF(−)^2)^	AAF(+)^2)^	AAF(−)^2)^	AAF(+)^2)^		AAF	LPS	AAF×LPS
Spleen								
21 d	0.80	0.90	1.12	1.08	0.09	0.75	0.02	0.48
35 d	1.17	1.33	2.30	1.42	0.46	0.30	0.01	0.05
Bursa fabricius								
21 d	2.62	2.55	2.63	2.39	0.17	0.42	0.71	0.68
35 d	1.96	2.43	2.07	2.37	0.17	0.05	0.87	0.66
Thymus								
21 d	2.21	2.02	2.53	2.39	0.11	0.26	0.03	0.86
35 d	1.88	2.02	2.00	2.20	0.18	0.44	0.47	0.87
Liver								
21 d	26.33	27.91	27.17	27.83	0.52	0.12	0.50	0.48
35 d	21.73	23.52	23.11	23.78	1.05	0.36	0.32	0.42

AAF, *Artemisia Argyi* flavonoids; LPS, lipopolysaccharide; SEM, standard error of the mean.

Data represent means from 6 replicates per treatment.

1) LPS(−), birds were intraperitoneally injected with saline; LPS(+), birds were intraperitoneally injected with LPS.

2) AAF(−), basal diet; AAF(+), basal diet supplemented with 750 mg/kg AAF.

**Table 5:** Effects of *Artemisia Argyi* flavonoids on the serum cytokines of broilers challenged with lipopolysaccharide

Item (pg/mL)	LPS(−)^1)^		LPS(+)^1)^		SEM	p-value		
		
	AAF(−)^2)^	AAF(+)^2)^	AAF(−)^2)^	AAF(+)^2)^		AAF	LPS	AAF×LPS
21 d								
IL-1β	71.06	72.51	81.33	73.19	1.84	0.11	0.02	0.05
IL-6	3.55	2.96	4.45	4.03	0.21	0.04	<0.01	0.71
28 d								
IL-1β	69.01	72.14	67.85	67.96	4.90	0.77	0.63	0.78
IL-6	3.35	3.20	3.15	3.50	0.10	0.40	0.97	0.05
35 d								
IL-1β	114.89	115.04	140.99	127.92	5.92	0.31	<0.01	0.30
IL-6	4.44	4.89	11.83	5.67	0.78	0.07	<0.01	<0.01
42 d								
IL-1β	74.93	78.49	72.82	72.96	14.26	0.80	0.59	0.80
IL-6	4.01	3.94	3.81	3.66	0.38	0.79	0.61	0.94

Data represent means from 6 replicates per treatment.

AAF, *Artemisia Argyi* flavonoids; LPS, lipopolysaccharide; SEM, standard error of the mean; IL-1β, interleukin1β; IL-6, interleukin-6.

1) LPS(−), birds were intraperitoneally injected with saline; LPS(+), birds were intraperitoneally injected with LPS.

2) AAF(−), basal diet; AAF(+), basal diet supplemented with 750 mg/kg AAF.

**Table 6:** Effects of *Artemisia Argyi* flavonoids on the liver cytokines of broilers challenged with lipopolysaccharide

Item (pg/mL Prot.)	LPS(−)^1)^		LPS(+)^1)^		SEM	p-value		
		
	AAF(−)^2)^	AAF(+)^2)^	AAF(−)^2)^	AAF(+)^2)^		AAF	LPS	AAF×LPS
21 d								
IL-1β	9.32	10.61	17.59	11.23	1.13	0.09	0.01	0.02
IL-6	0.80	0.79	1.77	1.35	0.16	0.46	<0.01	0.28
35 d								
IL-1β	11.81	13.66	30.78	12.06	2.83	0.01	0.02	<0.01
IL-6	0.80	0.82	2.79	1.20	0.20	<0.01	<0.01	<0.01

Data represent means from 6 replicates per treatment.

AAF, *Artemisia Argyi* flavonoids; LPS, lipopolysaccharide; SEM, standard error of the mean; IL-1β, interleukin-1β; IL-6, interleukin-6.

1) LPS(−), birds were intraperitoneally injected with saline; LPS(+), birds were intraperitoneally injected with LPS.

2) AAF(−), basal diet; AAF(+), basal diet supplemented with 750 mg/kg AAF.

**Table 7:** Effects of *Artemisia Argyi* flavonoids on the spleen cytokines of broilers challenged with lipopolysaccharide

Item (pg/mL Prot.)	LPS(−)^1)^		LPS(+)^1)^		SEM	p-value		
		
	AAF(−)^2)^	AAF(+)^2)^	AAF(−)^2)^	AAF(+)^2)^		AAF	LPS	AAF×LPS
21 d								
IL-1β	14.05	11.91	19.01	14.68	0.84	0.02	<0.01	0.32
IL-6	1.22	1.03	1.67	1.24	0.14	0.05	0.05	0.45
35 d								
IL-1β	7.35	7.60	13.48	9.75	0.67	0.05	<0.01	0.03
IL-6	0.86	0.73	1.26	0.95	0.07	0.05	<0.01	0.30

Data represent means from 6 replicates per treatment.

AAF, *Artemisia Argyi* flavonoids; LPS, lipopolysaccharide; SEM, standard error of the mean; IL-1β, interleukin-1β; IL-6, interleukin-6.

1) LPS(−), birds were intraperitoneally injected with saline; LPS(+), birds were intraperitoneally injected with LPS.

2) AAF(−), basal diet; AAF(+), basal diet supplemented with 750 mg/kg AAF.

## References

[1] Li R, Song ZH, Zhao JF (2018). Dietary L-theanine alleviated lipopolysaccharide-induced immunological stress in yellow-feathered broilers. Anim Nutr.

[2] Kamboh AA, Hang SQ, Khan MA, Zu WY (2016). *In vivo* immunomodulatory effects of plant flavonoids in lipopolysaccharide-challenged broilers. Animal.

[3] NghoNjuyi NW, Tiambo CK, Kimbi HK, Manka’a CAN, Juliano RS, Lisita F (2015). Efficacy of ethanolic extract of carica papaya leaves as a substitute of sulphanomide for the control of coccidiosis in KABIR Chickens in cameroon. J Anim Health Prod.

[4] Shen YB, Piao XS, Kim SW, Wang L, Liu P (2010). The effects of berberine on the magnitude of the acute inflammatory response induced by *Escherichia coli* lipopolysaccharide in broiler chickens. Poult Sci.

[5] Kamboh AA, Zhu WY (2014). Individual and combined effects of genistein and hesperidin on immunity and intestinal morphometry in lipopolysacharide-challenged broiler chickens. Poult Sci.

[6] Hager-Theodorides AL, Goliomytis M, Delis S, Deligeorgis S (2014). Effects of dietary supplementation with quercetin on broiler immunological characteristics. Anim Feed Sci Technol.

[7] Kamboh AA, Memon AM, Mughal MJ, Memon J, Bakhetgul M (2018). Dietary effects of soy and citrus flavonoid on antioxidation and microbial quality of meat in broilers. J Anim Physiol Anim Nutr.

[8] Li YJ, Guo Y, Yang Q (2015). Flavonoids casticin and chrysosplenol D from *Artemisia annua* L. inhibit inflammation *in vitro* and *in vivo*. Toxicol Appl Pharmacol.

[9] Amat N, Upur H, Blazekovic B, Blaekovi B (2010). *In vivo* hepatoprotective activity of theaqueous extract of *Artemisia absinthium* L. against chemically and immunologically induced liver injuries in mice. J Ethnopharmacol.

[10] Zhang PF, Shi BL, Yan SM (2017). Relieving effect of *Artemisia argyi* aqueous extract on immune stress in broilers. J Anim Physiol Anim Nutr.

[11] Wang X, Huang H, Ma X (2018). Anti-inflammatory effects and mechanism of the total flavonoids from *Artemisia scoparia* Waldst. et kit. *in vitro* and *in vivo*. Biomed Pharmacother.

[12] Wang DF, Zhang NY, Peng YZ, Qi DS (2010). Interaction of zearalenone and soybean isoflavone on the development of reproductive organs, reproductive hormones and estrogen receptor expression in prepubertal gilts. Anim Reprod Sci.

[13] Klaudia C, Alina W (2015). The influence of enrofloxacin, florfenicol, ceftiofur and *E. coli* LPS interaction on T and B cells subset in chicks. Vet Res Commun.

[14] Li Y, Zhang HZ, Chen YP (2015). *Bacillus amyloliquefaciens* supplementation alleviates immunological stress in lipopolysaccharide-challenged broilers at early age. Poult Sci.

[15] Zheng XC, Wu QJ, Song ZH (2016). Effects of Oridonin on growth performance and oxidative stress in broilers challenged with lipopolysaccharide. Poult Sci.

[16] Zhou Y, Mao S, Zhou M (2019). Effect of the flavonoid baicalein as a feed additive on the growth performance, immunity, and antioxidant capacity of broiler chickens. Poult Sci.

[17] Rasouli E, Jahanian R (2015). Improved performance and immunological responses as the result of dietary genistein supplementation of broiler chicks. Animal.

[18] Ouyang KH, Xu M, Jiang Y, Wang WJ (2016). Effects of alfalfa flavonoids on broiler performance, meat quality and gene expression. Can J Anim Sci.

[19] Ma JS, Chang WH, Liu GH (2015). Effects of flavones of sea buckthorn fruits on growth performance, carcass quality, fat deposition and lipometabolism for broilers. Poult Sci.

[20] Chen HM, Hsu JH, Liou SF (2014). Baicalein, an active component of *Scutellaria baicalensis* Georgi, prevents lysophosphatidylcholine-induced cardiac injury by reducing reactive oxygen species production, calcium overload and apoptosis via MAPK pathways. BMC Complement Altern Med.

[21] Yang YF, Zhao LL, Shao YX (2019). Effects of dietary graded levels of cinnamon essential oil and its combination with bamboo leaf flavonoid on immune function, antioxidative ability and intestinal microbiota of broilers. J Integr Agric.

[22] Yang L, Liu G, Zhu X, Luo Y, Shang Y, Gu XL (2019). The anti-inflammatory and antioxidant effects of leonurine hydrochloride after lipopolysaccharide challenge in broiler chicks. Poult Sci.

[23] Wu QJ, Wang YQ, Qi YX (2017). Influence of procyanidin supplementation on the immune responses of broilers challenged with lipopolysaccharide. Anim Sci J.

[24] Klasing KC, Korver DR (1997). Leukocytic cytokines regulate growth rate and composition following activation of the immune system. J Anim Sci.

[25] Wu X, Cao W, Jia G (2017). New insights into the role of spermine in enhancing the antioxidant capacity of rat spleen and liver under oxidative stress. Anim Nutr.

[26] Redmond SB, Tell RM, Coble D (2010). Differential splenic cytokine responses to dietary immune modulation by diverse chicken lines. Poult Sci.

[27] Liu SD, Song MH, Yun W, Lee JH, Kim HB, Cho JH (2019). Effect of carvacrol essential oils on immune response and inflammation-related genes expression in broilers challenged by lipopolysaccharide. Poult Sci.

[28] Esmaeil N, Anaraki SB, Gharagozloo M, Moayedi B (2017). Silymarin impacts on immune system as an immunomodulator: One key for many locks. Int Immunopharmacol.

[29] Zhang XH, Zhong X, Zhou YM, Wang GQ, Du HM, Wang T (2010). Dietary RRR-α-tocopherol succinate attenuates lipopolysaccharide-induced inflammatory cytokines secretion in broiler chicks. Br J Nutr.

[30] Zeinali M, Rezaee SA, Hosseinzadeh H (2017). An overview on immunoregulatory and anti-inflammatory properties of chrysin and flavonoids substances. Biomed Pharmacother.

